# The Safety and Immunogenicity of Trivalent Inactivated Influenza Vaccination: A Study of Maternal-Cord Blood Pairs in Taiwan

**DOI:** 10.1371/journal.pone.0062983

**Published:** 2013-06-06

**Authors:** Shin-Yu Lin, En-Tzu Wu, Chia-Hui Lin, Ming-Kwang Shyu, Chien-Nan Lee

**Affiliations:** 1 Department of Obstetrics and Gynecology, National Taiwan University Hospital, Hsin Chu branch, Hsin Chu, Taiwan; 2 Graduate Institute of Clinical Medicine, National Taiwan University College of Medicine, Taipei, Taiwan; 3 Department of Obstetrics and Gynecology, National Taiwan University Hospital, Taipei, Taiwan; University of Chieti, Italy

## Abstract

**Background:**

There are little data about adverse effects and immunogenicity of flu vaccine in Asian pregnant women.

**Methods:**

This prospective trial (NCT01514708) enrolled 46 pregnant women who received a single intramuscular dose of trivalent flu vaccine (AdimFlu-S®) containing 15 mcg of hemagglutinin for each strain/0.5 mL from influenza A (H1N1), influenza A (H3N2), and influenza B after the first trimester. Blood samples were collected at day 0 and 28 after vaccination, and at delivery. Cord blood was also collected. Hemagglutination inhibition (HAI) assays were performed to determine seroprotection and seroconversion rates and fold increase in the HAI geometric mean titer (GMT).

**Results:**

Twenty-eight days after vaccination the seroprotection rate against H1N1, H3N2, and influenza B was 91.3%, 84.8% and 56.5%, respectively. The GMT fold increase was 12.8, 8.4, and 4.6 for H1N1, H3N2, and influenza B, respectively. At delivery, both the seroprotection rate (86.4%, 68.2%, and 47.7%) and GMT fold increase (9.4, 5.7 and 3.8) were slightly lower than day 28. The seroprotection rate and GMT fold increase in maternal and cord blood samples were comparable. No significant adverse effects were detected.

**Conclusions:**

Trivalent flu vaccine induces a strong immune response in pregnant women and their infants without adverse effects.

**Trial Registration:**

Clinical Trials. gov NCT01514708

## Introduction

Influenza virus infection is a common cause of hospitalization and death, and worldwide the mortality from seasonal influenza virus infection is estimated to be 250,000 to 500,000 persons per year [1]. Pregnant women are at increased risk for influenza-associated illness and death [2], [3]. Neuzil et al. [4] quantified influenza-related serious morbidity in pregnant women during predefined influenza seasons and found that the risk of influenza related acute cardiopulmonary conditions was higher in pregnant women than in nonpregnant and postpartum women. In addition, the authors reported that the odds ratio (OR) was increased about 3-fold for women at 37–42 weeks' gestation as compared with those at 14–20 weeks' gestation. Another study reported that pregnant women with asthma were at high risk for hospitalization during the flu season [5]. Furthermore, influenza infection in young infants often prompts hospitalization and can predispose the infants to pneumonia or death, especially in infants under the age of 6 months [1], [6].

Since no influenza vaccine has been licensed for use in infants less than 6 months of age, and the mortality and morbidity of influenza infection is high in pregnant women, maternal influenza immunization is a promising resolution for protecting both mothers and infants [7]–[9]. Influenza vaccine using inactivated virus as the antigen had been proven safe for pregnant women and the fetus [10]. A study that included more than 2,000 pregnant women who received an inactivated virus influenza vaccine revealed no fetal malignancies [11]. Deinard et al. [12] demonstrated no teratogenicity in the infants of 189 pregnant women immunized with the influenza A/New Jersery/8/76 virus vaccine. A retrospective study of pregnant women who received the influenza vaccine in the second or third trimester of gestation revealed no serious adverse effects in the perinatal period or in infants during the first 6 months of live [13]. Other studies have also confirmed no adverse effects in infants when their mothers are administered inactivated virus influenza vaccines during the antepartum period [14], [15].

Immunization of pregnant women for influenza has also been shown to provide benefits for the infant. Zaman et al. [16] reported that administrating influenza vaccine in the third trimester could reduce influenza illnesses by 63% in infants up to 6 months of age, and avoid approximately 1/3 of respiratory illness in mothers and young infants. It has been demonstrated that vaccination or pregnant women with inactivated H1N1 virus can elicit an antibody response typically associated with protection against influenza infection, and result in efficient transplacental transfer of antibody to the newborn [17]–[21].

The World Health Organization (WHO) recommends that all pregnant women be immunized during the influenza season [22], while the United States (US) Centers for Disease Control and Prevention (CDC) also recommend that women who are or will be pregnant during the flu season get the flu vaccine [23]. The American College of Obstetricians and Gynecologists (ACOG) concurs with this recommendation [24]. In Canada and many European countries vaccinating healthy pregnant women is also recommended [2], [25]. The Advisory Committee on Immunization Practices in Taiwan recommends and prioritizes pregnant women to receive influenza vaccination, regardless of the stage of pregnancy. We previously conducted a retrospective study to evaluate the incidence, nature, and seriousness of adverse drug reactions (ADRs) occurring after AdimFlu-S® influenza A (H1N1) vaccination in pregnant women in Taiwan, and reported that influenza A vaccination during pregnancy did not lead to a higher incidence rate of maternal or fetal adverse events [15]. Evaluation of the safety and immunogenicity of influenza vaccine in pregnant women may provide useful information to reduce the hospitalization rate of pregnant women during influenza seasons. The aim of this study was to evaluate the safety, immunogenicity, and placental transfer of vaccine-specific antibody of the trivalent influenza vaccine, AdimFlu-S®, in pregnant women in Taiwan.

## Methods

### Vaccine composition

The study vaccine, AdimFlu-S® prepared by Adimmune (Taichung, Taiwan), is a trivalent, inactivated, split-virion influenza vaccine containing hemagglutinin (HA) from each of 3 reassortant viruses, including A/California/7/2009 (H1N1), A/Perth/16/2009 (H3N2), and B/Brisbane/60/2008 strains recommended by the WHO [26]. The viruses were propagated in embryonated chicken eggs, and after incubation the virus-containing allantoic fluid was collected and sucrose density gradient centrifugation was performed to isolate and concentrate the virus particles. The virus particles were then lysed by ether and the HA fraction was recovered, inactivated by formalin, and diluted with phosphate buffered solution. The vaccine containing 15 mcg of HA for each strain/0.5 mL, thimerosal (≤0.005 mg/mL), polysorbate 80 (≤0.1 μL/mL), and formalin (≤0.1 μL/mL) was injected intramuscularly into the upper arm. The Adimmune Corporation provided the vaccine, AdimFlu-S®, for this study, but had no role in the design or conduction of the study, analysis of the data, or preparation of this report.

### Study design and subjects

A prospective clinical trial was begun in December 2011 in Taipei, Taiwan, to evaluate safety and immunogenicity of the AdimFlu-S® influenza vaccine in pregnant women (Clinical trial registration name: The safety and immune response to influenza vaccination in pregnant women; number: NCT01514708; protocol available at Protocol S1). The study was approved by the Institutional Review Board of National Taiwan University Hospital (201109006MA, Text S1). CONSORT 2010 checklist of the study is available at Checklist S1. Pregnant women ≥18 years of age who were more than 20 weeks' gestation were screened for eligibility, and written informed consent was obtained. The fetal anatomic scan would be performed before 20 weeks' gestation so we chose the weeks' gestation cut-off of 20 weeks as an inclusion criterion for study enrollment. Exclusions included previous complicated pregnancy, preterm delivery, spontaneous or medical abortion, gestational diabetes mellitus (GDM), pregnancy induced hypertension (PIH), preeclampsia, received influenza vaccine within the prior 6 months, hypersensitivity to eggs or thimerosal, Guillain-Barré Syndrome, common cold or nasal congestion within the past 72 hours, influenza-like illness, immunodeficiency, and treatment with an investigational drug or device, immune-suppressive therapy, and/or blood transfusion within the prior 3 months. Before vaccination, a 10 mL venous blood sample was taken from each eligible subject for determining baseline serology. Each subject received 1 dose of the vaccine (0.5 mL) by intramuscular injection into the upper arm. After vaccination, subjects were asked to stay for 30 minutes to observe their immediate response. Follow-up blood samples were taken 4 weeks post immunization. Blood samples were also obtained from each subject immediately prior to delivery, and a cord blood sample was collected at the time of delivery.

The primary endpoint our this study is to evaluate the immune response of the three vaccine viral strains by calculation of the geometric mean titers (GMT) of anti-HA antibodies, geometric means of post- to pre-vaccination antibody titer ratios, seroprotection rates, and seroconversion rates. The secondary endpoint was to evaluate the incidence rate of pre-specified adverse events and all serious/non-serious adverse events. Safety data consisted of reactogenicity, serious and non-serious adverse events reported by the subject or observed by the investigator within 4 weeks after the vaccination, including 7 days after the dose of study vaccine. Vital signs were performed at baseline and 4 weeks post-vaccination. Each subject was instructed to record symptoms once each day on a diary card for 7 days. Serious adverse events during the first 4 weeks were documented. The selections of the events were collected systematically based on events expected to occur with wild-type influenza infection. The events included fever (≥38.0°C), runny nose or nasal congestion, cough, sore throat, muscle aches, headache, vomiting, nausea and malaise. Furthermore, the local (injection site) reactions were also be evaluated, including soreness/pain, swelling, redness, ecchymosis and limitation of arm motion.

Adverse events of special interest, including influenza (with positive test for the presence of influenza), pneumonia (with positive X-ray or at least three of the six clinical criteria suggestive of pneumonia – reduced breathing frequency, dull percussion, local crepitation, bronchophony, temperature ≥38°C and thorax pain), heart failure (requires confirmation by cardiologist or at least three of five symptoms suggested of heart failure – edema, increased central venous pressure, pleural signs, enlarged heart, and dyspnea), stroke (diagnosed by a specialist), exacerbation of chronic pulmonary disease and other respiratory illness with fever, and all serious adverse events and adverse events of special interest on both maternal subjects and their infants were monitored until 8 weeks after the delivery. The subjects were instructed to return according to their regular check-up schedule. The interval between the first and second blood sampling was at least 3 weeks, but not more than 5 weeks. It is usually considered that following vaccination, anti-HA antibody titers (measured by the hemagglutination-inhibition assay) peak 2–4 weeks post-vaccination in primed individuals but may peak 4 weeks or later in unprimed individuals or older adults [27]. It is suggested in the European Medicinal Agency guidance that the timing to take the blood sample is approximately 3 weeks after vaccination for the yearly clinical trials on influenza vaccine [28]. However we designed a 4-week duration for blood sampling to facilitate the process since 4-week is usually a regular clinical visit duration for pregnant women. Pregnancy outcomes including the type of delivery were recorded. Infant information including birth length, weight, sex, Apgar score at 1 min and 5 min, and other information specified in the medical chart were collected. The occurrence of adverse events was monitored for 8 weeks after delivery.

### Laboratory assays

Hemagglutination inhibition (HAI) assays was performed at the Adimmune Corporation designated central laboratory, and validated according to international standards. Antigens including A/California/7/2007, A/Perth/16/2009, and B/Brisbane/60/2008, were provided by Adimmune Corporation and were prepared by inactivating the whole virus with formalin. Reference antiserum to A/California/7/2009, A/Perth/16/2009, and B/Brisbane/60/2008 were obtained from the National Institute for Biological Standards and Control (NIBSC). Serum samples were treated with receptor-destroying enzymes to eliminate nonspecific hemagglutination inhibitors. Each sample was tested in duplicate an initial dilution of 1∶10. All laboratory personnel were blinded to the sample identity. For the purposes of calculation, samples with the titer of <1∶10 (negative) were assigned a titer of 1∶5.

### Statistical analysis

The 3 co-primary immunologic endpoints were seroprotection (HI titer >40), seroconversion (>4-fold increase in titer from baseline and post-vaccination HI titer >40 if the baseline titer was <10), and fold increase in geometric mean titer (GMT). Continuous variables were expressed as mean ± SD, while categorical variables were presented as number (percentage). The Mc'Nemar and binominal exact test were used to detect the change of seroprotection rate over time and compare the seroconversion rate with immunogenicity criteria of Committee for Proprietary Medicinal Products (CPMP), respectively; Wilcoxon signed rank test was used to detect the change of HAI titer over time. The Mc'Nemar exact test was used to detect any difference in seroprotection rate among maternal subjects and cord blood at delivery; Wilcoxon signed rank test was used to detect any difference in HAI titer among maternal subjects and cord blood at delivery.

The significance level α was set at 0.05. The statistical analysis was performed using SAS 9.3 (SAS Institute Inc., Cary, NC, USA). The sample size of this study was not based on power calculations. The sample size calculation was based on the expected precision of the results (extent of the 95% CI) and on the European recommendations for studies evaluating influenza vaccines to be used during each influenza season (at least 50 subjects per group). Therefore, 60 maternal subjects were planned to be recruited. This study was to demonstrate the AdimFlu-S® would induce adequate immune responses, and would be safe for pregnant women.

## Results

**Figure 1:**
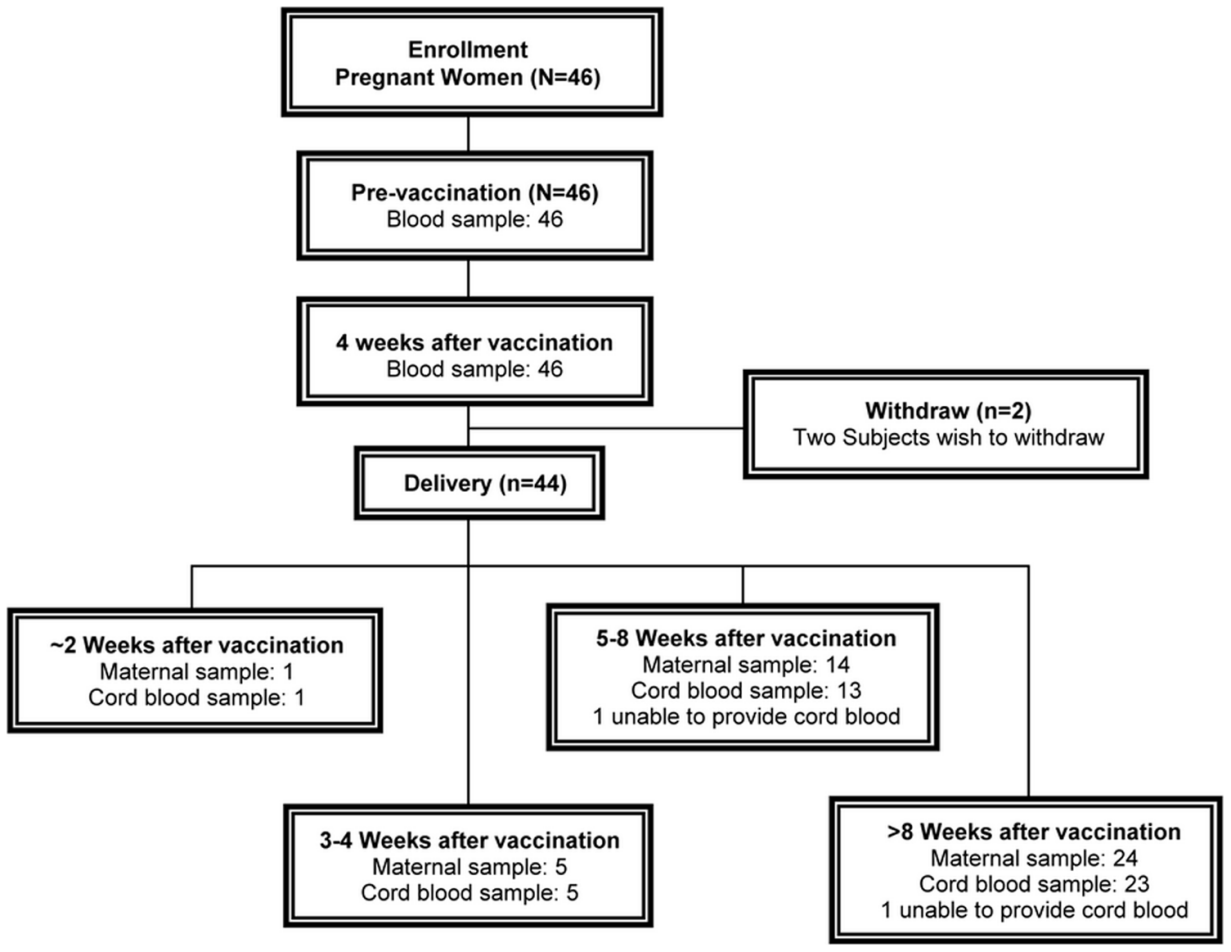
The trial profiles.

A total of 46 subjects with singleton pregnancies were enrolled, had baseline serology testing, and received the vaccination (Figure 1). Two subjects withdrew from the study after vaccination, and cord blood samples were not obtained from 2 subjects because they did not deliver the baby at the assigned hospital. The demographic, clinical, and obstetric characteristics of all 46 vaccinated pregnant subjects are summarized in Table 1. The mean age of the subjects was 33.0 years and the mean body mass index (BMI) was 25.3 kg/m^2^. None of the subjects reported a drinking history and 45 (97.8%) denied a history of smoking. Twenty-eight subjects (60.9%) reported symptoms of an illness within the prior 3 months, and the most commonly reported was abdominal pain (10.9%). In addition, 11 subjects (23.9%) had at least 1 concurrent medical condition. The mean gestational age at which the vaccine was administered was 29.0 weeks.

**Table 1:** Demographic, clinical, and obstetric characteristics of the 46 participants.

Age (y)	33.0±3.6
Weight (kg)	65.2±8.1
Height (cm)	160.8±5.4
Body mass index (kg/m^2^)	25.3±3.2
Smoking history	
Never	45 (97.8)
Quit smoking	1 (2.2)
Drinking history	0 (0.0)
Medical history within the past 3 months	
Abdominal pain	5 (10.9)
Constipation	3 (6.5)
Nasopharyngitis	3 (6.5)
Upper respiratory tract infection	3 (6.5)
Concurrent medical condition	
Cyanosis	1 (2.2)
Thalassemia	1 (2.2)
Goiter	1 (2.2)
Hyperthyroidism	1 (2.2)
Viral hepatitis carrier	1 (2.2)
Uterine leiomyoma	1 (2.2)
Hepatic hemangioma rupture	1 (2.2)
Placenta previa	1 (2.2)
Hemorrhagic ovarian cyst	1 (2.2)
Shortened cervix	1 (2.2)
Asthma	1 (2.2)
Nasal congestion	1 (2.2)
Gestational age at vaccination (wk)	29.0±4.7
Singleton	46 (100.0)
Age at birth of first child (y)	30.3±2.9
Number of pregnancies	
1	24 (52.2)
2	14 (30.4)
3	8 (17.4)
Previous delivery method*	
Vaginal delivery	11 (50.0)
Cesarean section	10 (45.5)
History of birth defect*	
None	20 (95.2)
Congenital heart disease	1 (4.8)

Data presented as mean ± standard deviation or number (percentage).

* Data only includes subjects with a prior delivery (*n* = 21).

Of the 46 subjects, it was the first pregnancy for 24 (52.2%), and their mean age was 30.3 years old. Among the subjects with previous pregnancies (*n* = 22), 11 had prior vaginal deliveries and 10 cesarean sections. One subject had a baby with congenital heart disease. No complications during previous pregnancies and/or deliveries were reported.

All 46 subjects had live births, and no birth defects were noted in any infants. The majority of the study subjects (93.2%) did not report any complications during delivery, and the mean gestational age at delivery was 38.8 weeks. One subject, P030, experienced premature rupture of membranes, placenta previa, and fetal distress, and 2 subjects, P017 and P031, experienced a prolonged labor during delivery. Twenty-seven subjects (61.4%) gave birth via unassisted vaginal delivery, 16 subjects (36.4%) received a cesarean section, and 1 subject (2.3%) required vacuum assistance during vaginal delivery. Among the 44 infants, 25 infants (56.8%) were boys and 19 (43.2%) were girls. The mean height was 49.6 cm, the mean head circumference was 33.9 cm, and the mean weight was 3175.3 g. The majority of the infants (95.5%) had an Apgar score of 9 at 1 min and 100% of the infants had an Apgar score ≥9 at 5 min.

Blood samples were obtained before and 4 weeks (28 days) after vaccination from 46 subjects. Samples were obtained from 44 subjects at the time of delivery, and 42 cord blood samples were collected. The baseline seropositive rate against H1N1, H3N2, and influenza B virus was 41.3%, 52.2%, and 43.5%, respectively. The baseline seroprotection rate against H1N1, H3N2, and influenza B virus was 21.7%, 21.7% and 8.7%. The baseline HAI GMT was 10.2±2.8, 10.8±2.4, and 8.7±2.4 for H1N1, H3N2, and influenza B virus, respectively. After vaccination, 44 subjects had detectable antibodies against all 3 influenza viruses, and the other 2 exhibited antibodies against 2 of the influenza viruses. As shown in Table 2, 4 weeks after vaccination the seroprotection rate of vaccinated subject against H1N1, H3N2, and influenza B virus was 91.3%, 84.8%, and 56.5%, respectively, and the seroconversion rate of vaccinated subjects was 67.4% for H1N1, 63.0% for H3N2, and 43.5% for influenza B virus. When compared with the baseline HAI GMT, the fold increase of GMT was 12.8±5.2 (GMT 129.6±3), 8.4±4.7 (GMT 90.9±3.8), and 4.6±3.2 (GMT 40±3.3) against H1N1, H3N3, and influenza B virus, respectively.

**Table 2:** Seroprotection rate, seroconversion rate, and HAI GMT of 46 subjects at day 28 after vaccination.

	Pre-vacci nation	Day 28	p-value
**Seroprotection Rate**			
A/California/7/2009 (H1N1)	10 (21.7)	42 (91.3)	<0.0001^a^ *
A/Perth/16/2009 (H3N2)	10 (21.7)	39 (84.8)	<0.0001^a^ *
B/Brisbane/60/2008	4 (8.7)	26 (56.5)	<0.0001^a^ *
**Seroconversion Rate**			
A/California/7/2009 (H1N1)	NA	31 (67.4)	<0.0001^b^ *
A/Perth/16/2009 (H3N2)	NA	29 (63.0)	0.0027^b^ *
B/Brisbane/60/2008	NA	20 (43.5)	0.7342^b^
**HAI GMT**			
A/California/7/2009 (H1N1)	10.2±2.8	129.6±3.0	<0.0001^c^ *
A/Perth/16/2009 (H3N2)	10.2±2.4	90.9±3.8	<0.0001^c^ *
B/Brisbane/60/2008	8.7±2.4	40.0±3.3	<0.0001^c^ *

Data presented as mean ± standard deviation or number (percentage).

a: Mc'Nemar exact test for the change of response over time.

b: Comparison between Seroconversion rate and immunogenicity criteria of Committee for Proprietary Medicinal Products (CPMP) was analyzed by Binomial Exact test.

c: Wilcoxon signed rank test for the change of HAI titer over time.

* : statistically significant (p<0.05).

NA, not applicable.

The immune response of vaccinated subjects at the time of delivery was determined. One subject gave birth within 2 weeks after vaccination and was found to have seroprotection against the 3 influenza viruses. Five subjects gave birth within 3 to 4 weeks after vaccination with a seroprotection rate of 80.0% against H1N1, 100.0% against H3N2, and 60.0% against influenza B. Fourteen subjects gave birth to within 5 to 8 weeks after vaccination with seroprotection rate of 85.7% against H1N1, 85.7% against H3N2, and 28.6% against influenza B. Twenty-four subjects gave birth more than 8 weeks after vaccination with a seroprotection rate of 87.5% against H1N1, 50.0% against H3N2, and 54.2% against influenza B. The seroprotection rates of all subjects (n = 44) at the time of delivery were 86.4%, 68.2%, and 47.4% against H1N1, H3N2, and influenza B virus, respectively.

The seroconversion rates at delivery were similar to the seroprotection rates (Table 3). The seroconversion rate of all vaccinated subjects (*n* = 44) at the delivery was 63.6%, 50% and 40.9% against H1N1, H3N2 and influenza B virus. The HAI GMT of all vaccinated subjects (*n* = 44) at delivery was 92.2, 59.3 and 31.6 against H1N1, H3N2 and influenza B virus, respectively.

**Table 3:** Seroprotection rate, seroconversion rate, and HAI GMT of 44 subjects at delivery.

	Pre-Vaccination (N = 44)	Delivery (N = 44)	p-value	0–2 weeks (n = 1)	3–4 weeks (n = 5)	5–8 weeks (n = 14)	>8 weeks (n = 24)
**Seroprotection Rate**							
A/California/7/2009 (H1N1)	9 (20.5)	38 (86.4)	<0.0001^a^ *	1 (100)	4 (80.0)	12 (85.7)	21 (87.5)
A/Perth/16/2009 (H3N2)	9 (20.5)	30 (68.2)	<0.0001^a^ *	1 (100)	5 (100.0)	12 (85.7)	12 (50.0)
B/Brisbane/60/2008	3 (6.8)	21 (47.7)	<0.0001^a^ *	1 (100)	3 (60.0)	4 (28.6)	13 (54.2)
**Seroconversion Rate**							
A/California/7/2009 (H1N1)	NA	28 (63.6)	0.0026^ b^ *	1 (100)	3 (60.0)	6 (42.9)	18 (75.0)
A/Perth/16/2009 (H3N2)	NA	22 (50.0)	0.2314^ b^	1 (100)	4 (80.0)	8 (57.1)	9 (37.5)
B/Brisbane/60/2008	NA	18 (40.9)	1.0000^ b^	1 (100)	3 (60.0)	3 (21.4)	11 (45.8)
**HAI GMT**							
A/California/7/2009 (H1N1)	9.8±2.7	92.2±3.2	<0.0001^c^ *	1280.0	183.8±4.5	69.0±2.8	84.8±2.9
A/Perth/16/2009 (H3N2)	10.5±2.4	59.3±3.9	<0.0001^c^ *	640.0	183.8±2.5	69.0±3.3	38.9±3.7
B/Brisbane/60/2008	8.3±2.1	31.6±3.3	<0.0001^c^ *	40.0	30.3±3.2	22.1±3.4	38.9±3.4

Two subjects, P005 and P039, who did not deliver at the study site were not included in this analysis. Data presented as mean±standard deviation or number (percentage).

a: Mc'Nemar exact test for the change of response over time.

b: Comparison between Seroconversion rate and immunogenicity criteria of Committee for Proprietary Medicinal Products (CPMP) was analyzed by Binomial Exact test.

c: Wilcoxon signed rank test for the change of HAI titer over time.

* : statistically significant (p<0.05).

Note: Periods were defined as follows: 0–2 weeks, delivery within 20 days after vaccination; 3–4 weeks, delivery at day 21 to day 34; 5–8 weeks, delivery at day 35 to 63; >8 weeks, delivery day 64 or later.

A summary of the seroprotection rates of cord blood samples (*n* = 42) is presented in Table 4. Cord blood samples and maternal blood samples at delivery exhibited similar seroprotection rates and GMT against the 3 influenza viruses, except that values of GMT in subjects who received the vaccine more than 5 weeks prior to delivery were higher in cord blood samples than in maternal samples.

**Table 4:** Seroprotection rate and HAI GMT of cord blood samples.

	Delivery (N = 42)	p-value	0–2 weeks (n = 1)	3–4 weeks (n = 5)	5–8 weeks (n = 13)	>8 weeks (n = 23)
**Seroprotection Rate**						
A/California/7/2009 (H1N1)	37 (88.1)	1.0000^a^	1 (100)	5 (100.0)	11 (84.6)	20 (87.0)
A/Perth/16/2009 (H3N2)	30 (71.4)	0.6875^a^	1 (100)	4 (80.0)	11 (84.6)	14 (60.9)
B/Brisbane/60/2008	20 (47.6)	1.0000^ a^	0 (0.0)	2 (40.0)	7 (53.8)	11 (47.8)
**HAI GMT**						
A/California/7/2009 (H1N1)	137.9±3.8	0.0028^b^ *	80.0	278.6±3.1	129.3±3.4	125.7±4.3
A/Perth/16/2009(H3N2)	74.9±4.0	0.1096^ b^	640.0	121.3±3.9	93.9±3.5	54.1±4.1
B/Brisbane/60/2008	35.1±4.0	0.3830^ b^	20.0	23.0±6.4	29.0±2.8	43.8±4.5

Data presented as mean±standard deviation or number (percentage).

a: Mc'Nemar exact test for the difference of seroprotection rate among maternal subjects and cord blood at delivery.

b: Wilcoxon signed rank test for the difference in HAI titer among maternal subjects and cord blood at delivery.

* : Statistically significant (p<0.05).

The mean investigated periods were 124.07 days (range: 72–176 days, standard deviation 30.57). Local and systemic reactions that occurred during the first 7 days after vaccination are summarized in Table 5. Thirty-four subjects (73.9%) had at least 1 local event. The most local event was injection-site pain (69.6%, *n* = 32), redness (32.6%, *n* = 15), and swelling (28.3%, *n* = 13). The majority of local events were mild; only 3 subjects reported moderate pain and 1 subject reported moderate swelling. Twenty-six subjects (56.5%) reported at least 1 systemic event after vaccination. The most common systemic event was malaise (43.5%, *n* = 20), while other common events were muscle aches (26.1%, *n* = 12), cough (26.1%, *n* = 12), and nasal congestion (23.9%, *n* = 11). The majority of reported systemic events were mild; 2 subjects reported moderate malaise, 2 reported moderate muscle aches, 2 reported moderate nasal congestion, and 1 reported moderate nausea.

**Table 5:** Adverse events occurring within 7 days after vaccination in 46 subjects.

	N (%)	Mild	Moderate	Severe
**Local events**				
Any	34 (73.9)			
Pain	32 (69.6)	29	3	0
Swelling	13 (28.3)	12	1	0
Redness	15 (32.6)	15	0	0
Ecchymosis	0 (0.0)	0	0	0
Decreased limb mobility	4 (8.7)	4	0	0
**Systemic events**				
Any	25 (56.5)			
Fever (≥38°C)	0 (0.0)	0	0	0
Nasal congestion	11 (23.9)	9	2	0
Cough	12 (26.1)	12	0	0
Sore throat	6 (13.0)	6	0	0
Muscle aches	12 (26.1)	10	2	0
Headache	5 (10.9)	5	0	0
Nausea	4 (8.7)	3	1	0
Vomiting	6 (13.0)	6	0	0
Malaise	20 (43.5)	18	2	0
Eye redness	2 (4.3)	2	0	0
Chest tightness	5 (10.9)	5	0	0
Respiratory distress	3 (6.5)	3	0	0
Face edema	1 (2.2)	1	0	0

Data presented as number (percentage).

Seven serious adverse events were reported by 5 subjects and included postpartum hemorrhage, premature delivery, gestational hypertension, and premature uterine contractions. The independent adjudication committee considered none of the events to be related to the vaccine. No serious adverse events were reported in any neonate, and no maternal or infant deaths occurred.

## Discussion

It is recommended that all women who will be pregnant during influenza season receive inactivated influenza vaccine at any point in gestation by The Centers for Disease Control and Prevention's Advisory Committee on Immunization Practices (ACIP) and The American College of Obstetricians and Gynecologists' Committee on Obstetric Practice [29]. However, published data of the maternal immunogenicity of influenza vaccines were mainly from the United States and Europe. To the best of our knowledge, ours is the first published trial to evaluate both maternal immune response and neonate seroprotection from a single dose of trivalent influenza vaccine in pregnant women in Asia. In this prospective study, we demonstrated that pregnant women receiving the trivalent influenza vaccine produce antibodies sufficient to provide protection against influenza infection both in the mother and the newborn.

An HAI antibody titer of 1∶40 after vaccination is the current standard for licensure of influenza vaccines, and a widely accepted surrogate for protection against influenza infection [30]. In this study, women who were vaccinated had HAI GMTs above this threshold value at day 28 against H1N1, H3N2, and influenza B virus and at delivery against H1N1 and H3N2 virus, suggesting protection against these specific influenza strains. On the other hand, according to the Committee of Medicinal Products for Human Use (CHMP) guidance, at least 1 of 3 serological assessments (seroprotection, seroconversion, and an increase ratio of HAI titers) is necessary to meet the requirements for seasonal influenza vaccines. In this study, 28 days after vaccination the seroprotection and seroconversion rates and the increased ratio of HAI titers against influenza type A (H1N1 and H3N2) viruses and the seroconversion and the increase ratio in HAI titers against influenza type B were fully compliant with the CHMP criteria for seasonal influenza vaccines. These data support the clinical utility of the AdimFlu-S® vaccine.

Vaccine administration to pregnant women has been used to protect infants against infection in the first few months of life. Here, we examined transplacental antibody transfer following influenza vaccination. The seroprotection rate of cord blood correlated to that of the maternal samples at delivery, consistent with a study by Sumaya and Gibbs [31]. Administration of the vaccine to pregnant women resulted in detectable antibodies against H1N1 and H3N2 virus in umbilical cord venous blood with GMTs >1∶40, but no enough rise of antibodies against influenza B virus. This finding is consistent with previous studies of seasonal influenza vaccination [32], [33]. The finding that GMT titers of influenza B virus were lower than those of H1N1 and H3N2 might be the result of poor sensitivity of the ELISA assay used for the detection of influenza B virus antigen.

Our results showed that cord blood samples had higher mean HAI titers than the maternal samples at the time of delivery, a finding consistent with those of a previous trial in pregnant women [34]. In that study, a single dose of a monovalent 2009 H1N1 flu vaccine was administrated to pregnant women, and a high seroprotection rate was reported at both delivery (92%) and in cord blood (95%) samples and the HAI GMT was higher in cord blood samples (413.4, 95% confidence interval [CI] 297.6–574.2) than in maternal samples at delivery (275.3, 95% CI 208.3–363.9). These data indicate that maternal antibodies are transferred to and can protect infants from influenza virus infection during the first months of life.

The safety profile of AdimFlu-S®, particularly the frequency and the severity of local and systemic events, was consistent with that found in prior studies of seasonal flu vaccines [35]. In general, the local and systemic events were mild to moderate in severity, and no deaths of adverse events of special interest such as the optic neuritis, cranial neuropathy, brachial neuropathy, and Guillain-Barre syndrome (GBS) were reported in this study. The majority of study subjects (93.2%) did not report any complications during delivery, and those complications that did occur were deemed to be not related to the vaccine. All subjects had a live birth and no birth defects were reported. These data indicate that AdimFlu-S® is safe and well tolerated for pregnant women and their newborns.

This study has limitations that should be considered. There were a small number of subjects and we were unable to determine a correlation of antibody titers with time after vaccination. We did not enroll pregnant women at the first trimester of gestation; although influenza vaccination is recommended for pregnant women regardless of the trimester, patients have safety concerns with vaccination in the first trimester of pregnancy. Only healthy pregnant women were studied. The results cannot be applied to the pregnant women with co-morbidity. Lastly, we showed that antibody titers declined over time, consistent with previous reports [21], [34]. However, we did not collect long-term follow-up data and cannot determine if antibody titers fell to zero or if the mothers and infants developed influenza infections.

## Conclusion

This prospective study examined the immunogenicity, safety, and transplacental transmission of antibodies in pregnant women in Asia vaccinated with AdimFlu-S® and the results showed that AdimFlu-S® vaccination induces a strong immune response and is safe for pregnant women. Our data also indicate that maternal antibodies are transferred to the infants.

## Supporting Information

Checklist S1 **CONSORT Checklist.**
(DOC)

Protocol S1 **Trial protocol.**
(PDF)

Text S1 **The approval of the study by the Research Ethics Committee A of the National Taiwan University Hospital.**
(PDF)
