# Supplementary material for: The Safety and Immunogenicity of Trivalent Inactivated Influenza Vaccination: A Study of Maternal-Cord Blood Pairs in Taiwan
Source: PLoS One. 2013 Jun 6;8(6):e62983. doi: 10.1371/journal.pone.0062983 (PMC3675132; doi:10.1371/journal.pone.0062983)
Supplement: Protocol S1 — Trial protocol. (PDF) [file pone.0062983.s002.pdf]

Clinical Study Protocol

The safety and immune response to influenza vaccination in pregnant women

Study Protocol: FLU11T12P

Version: 1.0

Date: 2011-08-08

Sponsor: Adimmune Corporation

CONFIDENTIAL AND PROPRIETARY

This document contains confidential information and is solely the property of Adimmune Corporation. Acceptance of this document constitutes an agreement by the recipient that no unpublished information contained herein will be published or disclosed without the prior written approval of Adimmune Corporation. This document may be disclosed to the appropriate Institutional Review Boards (IRBs) with the stipulation that they (the IRBs) treat the information contained herein as the confidential property of Adimmune Corporation.

TABLE OF CONTENTS

Table of Contents i

List of Abbreviations and Definitions iii

1. Introduction 1

2. Study Objectives 3

2.1 Primary Objective 3

2.2 Secondary Objective 3

2.3 Outcome Measure 3

3. Study Design 4

3.1 Study Design 4

3.2 Number of Subjects 4

3.3 Subject Selection Criteria 4

3.4 Discontinuation for Individual Subjects 5

4. Study Treatment 6

4.1 Details of Study Treatments 6

4.2 Administration and Dosage 7

4.3 Storage and Labeling 7

4.4 Concomitant Vaccinations and Medications 8

5. Study Procedures and Schedule 8

5.1 Study Procedure 8

5.2 Immunogenicity 9

5.3 Safety 10

5.4 Schedule 11

5.5 Serum Sample Randomization and Blinding 11

6. Adverse Events 12

7. Statistical Analysis 14
7.1 Sample Size Consideration 14
7.2 Study Population 15
7.3 Demographics 15
7.4 Analysis of Immunogenicity 15
7.5 Analysis of Safety 16
8. Ethical and Legal Aspects 16
8.1 Good Clinical Practice 16
8.2 Delegation of Investigator Responsibilities 16
8.3 Subject Information and Informed Consent 17
8.4 Confidentiality 17
8.5 Protocol Amendments 18
8.6 Approval of the Study Protocol and Amendments 18
9. Study Monitoring and Auditing 19
9.1 Study Monitoring 19
9.2 Source Documentation, Records Retention, and Availability 19
9.3 Source Data Verification and On-Site Audits 20
10. Reference 21

LIST OF ABBREVIATIONS AND DEFINITIONS

Abbreviation	Definition
ACIP	Advisory Committee on Immunization Practices
ACOG	The American College of Obstetricians and Gynecologists
AE	Adverse Events
BMI	Body Mass Index
CDC	Centers for Disease Control and Prevention
CI	Confidence Intervals
COPD	Chronic Obstructive Pulmonary Disease
CPMP	Committee for Proprietary Medicinal Products
CRF	Case Report Form
EC	Ethics Committee
EMA	European Medicines Agency
EU	European Union
GBS	Guillain-Barre syndrome
GCP	Good Clinical Practice
GDM	Gestational Diabetes Mellitus
GMT	Geometric Mean Titer
GMTR	Geometric Mean Titer Ratio
HA	Hemagglutinin
HAI	Hemagglutinaton Inhibition
ICH	International Conference on Harmonisation
IEC	Independent Ethics Committee
IRB	Institutional Review Board
MedDRA	Medical Dictionary for Regulatory Activities
RNA	Ribonucleic acid
SAE	Serious Adverse Events
SOC	System Organ Class
URI	Upper respiratory illness
WHO	World Health Organization

1. INTRODUCTION

Influenza, commonly called "the flu," is an illness caused by RNA viruses that infect the respiratory tract of many animals, birds, and humans. In most people, the infection results in the person getting fever, cough, headache, and malaise (tired, no energy); some people also may develop a sore throat, nausea, vomiting, and diarrhea. The majority of individuals has symptoms for about one to two weeks and then recovers with no problems. However, compared with most other viral respiratory infections, such as the common cold, influenza (flu) infection can cause a more severe illness with a mortality rate (death rate) of about 0.1% of people who are infected with the virus.

Pregnant women, even those who are healthy, can become very sick if they get the flu (influenza). For example, one large study reported in 1998 documented the serious impact that flu can have during pregnancy. [1] This study examined the effect of the flu on pregnant women over 17 previous flu seasons. It found that during the flu season, pregnant women in the third trimester were just as likely to be hospitalized for heart or lung problems as women with serious, chronic medical conditions who were not pregnant. The risk increased the farther along the women were in their pregnancies. Healthy women at 37 to 42 weeks were almost five times as likely to be admitted to the hospital during the flu season for heart or lung problems as women who were one to six months postpartum. Another study showed that pregnant women with asthma in particular were at high risk for hospitalization during the flu season. [2]

Because of these findings, the U.S. Centers for Disease Control and Prevention (CDC) recommend that women who are or will be pregnant during the flu season get the flu vaccine. [3] The American College of Obstetricians and Gynecologists (ACOG) concurs with this recommendation. [4]

Experts believe that the flu vaccine will provide protection against the flu in pregnant women (as it does in healthy adults) although earlier observational research could not confirm this. [5] However, a more recent experimental study from Bangladesh suggests protection against the flu for both the mother and infant when the mother was vaccinated against the flu. [6]

Because the flu vaccine is an inactivated vaccine (containing no live virus), vaccine experts believe it to be safe for pregnant women. An early study of the flu vaccine in more than 2,000

pregnant women revealed no excess malignancies in the fetus. [7] A similar but smaller study revealed no harmful effects of the flu vaccine on the fetus or the mother. [8] More recent research found no serious adverse effects from the flu vaccine in the perinatal period or during the first six months of infant life. [9] Although the numbers of patients in these studies are relatively small, these results are reassuring.

According to the previous result, in the season of 2010-2011, the adults (aged between 20 and 60) had the seroprotection rates (HAI titer ≥ 1:40) were 92.1% against influenza A/H1N1 virus, 85.7% against influenza A/H3N2 virus and 44.4% against influenza B virus 3 weeks after vaccination. The seroconversion rates were 79.4% against influenza A/H1N1 virus, 68.3% against influenza A/H3N2 virus and 28.6% against influenza B virus.

Moreover, 68.3% were females. Among these 43 female subjects, the seroprotection rates were 90.7% against influenza A/H1N1 virus, 88.4% against influenza A/H3N2 virus and 48.8% against influenza B virus 3 weeks after vaccination. The seroconversion rates were 79.1% against influenza A/H1N1 virus, 76.7% against influenza A/H3N2 virus and 34.9% against influenza B virus. Furthermore, 26 females was aged between 18~40 years old, inclusive. Among these 26 female subjects, the seroprotection rates were 92.3% against influenza A/H1N1 virus, 88.5% against influenza A/H3N2 virus and 52.8% against influenza B virus 3 weeks after vaccination. The HAI titer geometric mean fold rise was 18.8±4.9 against influenza A/H1N1 virus, 14.2±5.2 against influenza A/H3N2 virus and 5.2±4.0 against influenza B virus 3 weeks after vaccination. The seroconversion rates were 84.6% against influenza A/H1N1 virus, 80.8% against influenza A/H3N2 virus and 38.5% against influenza B virus.

Another trial on infants between 6 to 12 months old, 57 subjects were included to receive two vaccinations at 4 weeks apart. The seroprotection rate before vaccination were 8.8% against influenza A/H1N1, 10.5% against influenza A/H3N2 virus and 0.0% against influenza B virus. The mean HAI titer geometric before vaccination were 6.5±2.4 against influenza A/H1N1 virus, 8.2±4.6 against influenza A/H3N2 virus and 5.2±1.2 against influenza B virus.

A close observation of safety and immunogenicity of maternal subjects receiving an influenza vaccine may provide useful immunization strategy for preventing influenza. Consequently, this study aimed to provide immunogenicity and safety profile after receiving the study vaccine in pregnant women.

2. STUDY OBJECTIVES

The purpose of this study is to evaluate the influenza vaccine (AdimFlu-S) to determine vaccine safety in pregnant women and the body's immune response.

2.1 Primary Objective

- To evaluate the immune response of the three vaccine viral strains by calculation of the geometric mean titers (GMT) of anti-HA antibodies, geometric means of post- to pre-vaccination antibody titer ratios, seroprotection rates, and seroconversion rates.

The primary endpoint will be the seroprotection rate which is defined as the proportion of subjects with HAI titer ≥ 1:40. The other immunogenicity endpoints include seroconversion rate and geometric mean folds increase in HAI titer. Immunogenicity profile will be assessed before vaccination, 4 weeks after the vaccination and at the end of gestation period.

Moreover, the HAI titer of the cord blood will also be measured and compared with the maternal blood sample.

2.2 Secondary Objective

The secondary endpoint is to evaluate the incidence rate of pre-specified adverse events and all serious/non-serious adverse events.

2.3 Outcome Measure

- Number of participants with seroprotection against influenza A/H1N1, A/H3N2, B virus (Day 28, the end of gestation period).
- Number of participants with seroconversion against influenza A/H1N1, A/H3N2, B virus (Day 28, the end of gestation period).
- Geometric means of post- to pre-vaccination antibody titer ratios against influenza A/H1N1, A/H3N2, B virus (Day 28, the end of gestation period).
- Number of participants reporting solicited subjective local reactions within 7 days (Day 1-7) post vaccination.
- Number of participants reporting solicited subjective systemic reactions within 7 days (Day 1-7) post vaccination.

- Number of participants reporting maternal complications of pregnancy, labor and delivery.
- Number of participants reporting neonatal complications.
- Number of participants reporting vaccine-associated serious adverse events (SAEs) during the study.

3. STUDY DESIGN

3.1 Study Design

This is a prospective, single-arm, descriptive clinical study to observed the safety and immunogenicity profile of the use of AdimFlu-S (2011-2012 season) vaccine in pregnant female subjects. All eligible participates will receive one dose of vaccine (0.5 mL) by intramuscular injection into the upper arm. Blood serum prepared from blood samples will be collected from each maternal subject immediately prior to vaccination and 4 weeks after vaccination; moreover, blood sample at the end of the gestation period (delivery) and the cord blood sample will also be collected, if applicable. Anti-hemagglutinin (HA) antibody titers will be determined using the WHO hemagglutination inhibition reference technique and the analysis will be observer-blinded. Safety outcomes included immediate reactions at the time of vaccination, solicited local and systemic reactions for 7 days, unsolicited adverse events until the first post-vaccination serum collected, and serious adverse events and adverse events of special interest until 8 weeks after the delivery.

3.2 Number of Subjects

Sixty subjects will be enrolled..

3.3 Subject Selection Criteria

3.3.1. Inclusion Criteria

Subjects meeting all of the following criteria will be considered for admission to the study:

1. Pregnant female aged ≥ 18 years old.
2. Subject is pregnant for at least 3 months, inclusive.

3. Subject is willing and able to adhere to visit schedules and all study requirements.
4. Subject has read and signed the study-specific informed consent.

3.3.2. Exclusion criteria:

1. Subject with previous complicated pregnancy or preterm delivery, spontaneous or medical abortion;
2. Subject with history or concurrent high risk of dangerous gestation such as gestational diabetes mellitus (GDM), pregnant induced hypertension, or preeclampsia;
3. Subject received any influenza vaccine within the previous 6 months;
4. Subject has a history of hypersensitivity to eggs or egg protein or similar pharmacological effects to study vaccine;
5. Subject or her family has the history of Guillain-Barré Syndrome;
6. Subject has current upper respiratory illness (URI), including the common cold or nasal congestion within 72 hours;
7. Subject with influenza-like illness as defined by the presence of fever (temperature ≥38℃) and at least two of the following four symptoms: headache, muscle/joint aches and pains (e.g. myalgia/arthralgia), sore throat and cough;
8. Subject receive any treatment with an investigational drug or device, or participation in a clinical study, within 3 months before consent;
9. Subject has immunodeficiency or is under immunosuppressive treatment.
10. Subject received any vaccine within 1 week prior to study vaccination or expected to receive one within 1 week after study vaccination;
11. Subject received any blood products, including immunoglobulin, in the past 3 months before consent;
12. Subject has underlying condition in the investigators' opinion may interfere with evaluation of the vaccine.

3.4 Discontinuation for Individual Subjects

A discontinuation is a subject who enrolled in the study and did not received the study vaccine for any reason.

The subject is free to withdraw from the study for any reason and at any time without giving reason for doing so and without penalty or prejudice. The investigator is also free to terminate

a subject's involvement in the study at any time if the subject did not received the study vaccine.

Discontinuation reason for enrolled subjects may be, but not limited to, the following:

1. Withdrawal of consent
2. Did not received study vaccine
3. Lost to follow up

The study subjects withdrawing for any reason should have a post-vaccination visit including at least all safety assessments.

There will be no replacement of withdrawn subjects, regardless of the reason for withdrawal. All subjects who experience adverse event which is ongoing either at the time of withdrawal or at the completion of whole study visit, must be followed-up at appropriate time intervals until the adverse event is resolved or judged to be no longer clinically significant, or until they become chronic to the extent that they can be fully characterized.

4. STUDY TREATMENT

4.1 Details of Study Treatments

The vaccine used in this study is the inactivated split influenza vaccine, licensed in Taiwan under the name AdimFlu-S. It is a sterile suspension prepared from influenza viruses propagated in chicken embryos. After the incubation, the virus-containing allantoic fluid is collected and the sucrose density gradient centrifugation is performed to isolate and concentrate the virus particles. Ether is added to lyse the virus particles and the resulting hemagglutinin (HA) fraction is recovered, inactivated by formalin, and diluted with phosphate buffered solution to formulate a final solution containing the required amount of HA of the virus strain.

Each year's influenza vaccine contains three virus strains (two type A and one type B) representing the influenza viruses that are likely to circulate in Taiwan in the coming winter. The antigenic characteristics of current and emerging influenza virus strains provide the basis for selecting the strains included in each year's vaccine.

For the 2011-2012 season, the vaccine contains not less than 90 μg hemagglutinin (HA) per

mL. The vaccine is formulated by the following three strains: [13]

A/California/7/2009 (H1N1)	30 μg/mL
A/Perth/16/2009 (H3N2)	30 μg/mL
B/Brisbane/60/2008	30 μg/mL

The vaccine contains thimerosal ≤ 0.005 mg/mL as a preservative, formalin ≤ 0.1μL/mL and polysorbate 80 ≤ 0.1μL/mL as a stabilizer.

After shaking syringe well, AdimFlu-S is essentially clear and opalescent whitish color.

The influenza virus for AdimFlu-S, Inactivated Influenza Vaccine Trivalent Types A and B (Split), is propagated in eggs, and therefore, this vaccine should not be administered to anyone with a history of hypersensitivity (allergy) and especially anaphylactic reactions to eggs or egg products. It is also a contraindication to administer this vaccine to individuals known to be sensitive to thimerosal.

4.2 Administration and Dosage

Before administration of any vaccine, all appropriate precautions should be taken to prevent adverse reactions. This includes a review of the subject's history with respect to possible hypersensitivity to the vaccine or similar vaccine, determination of previous immunization history, and the presence of any contraindications to immunization, current health status, and a current knowledge of the literature concerning the use of the vaccine consideration.

In this single-dose study, the administration of the investigational product will be supervised by the investigator or sub-investigator. Each subject will receive 1 dose of vaccine (0.5 mL) by intramuscular injection into the upper arm. The injection will be given into the opposite arm from which blood was drawn.

Detail of the date of administration of study vaccine will be documented in the case report form.

4.3 Storage and Labeling

Labeling for syringe will meet all official regulations. All syringes will be supplied to the clinical study site in boxes that need to be stored between 2 and 8°C and do not freeze. The study vaccine will be stored under secure conditions. Product that has been exposed to

freezing should not be used.

4.4 Concomitant Vaccinations and Medications

No concomitant immunizations (for 7 days post-vaccination) are permitted. All concomitant medications taken during the first and second venous blood samples (4 weeks post-vaccination) collected should be recorded on the case report form. Nevertheless, immunization and immunotherapies for maternal subjects after vaccination and until the end of gestation period should be recorded.

5. STUDY PROCEDURES AND SCHEDULE

5.1 Study Procedure

Each potential female subject will be screened before the start of the study to determine their eligibility for participation. The following examination will be performed and documented for each subject:

- Demographics including age, height, weight, weeks of gestation and influenza vaccination at 2010-2011 season;
- Blood pressure and heart rate and ear temperature;
- Review and record all medical history including history of allergy to food, medication or blood transfusion; concurrent and prior medical conditions; and immune system disorder and smoking history.
- Review and record all obstetric history, including but not limited to the first gestational age at birth, the gravidity (the number of times a woman has been pregnant), history of previous complicated pregnancy or preterm delivery, spontaneous or medical abortion, and congenital anomaly.
- Review and record all medication taken in the past month;

Just prior to vaccination, a 10 mL venous blood sample will be taken from each eligible subject, for baseline titration of circulating anti-HA antibodies. Immediately thereafter, each subject will receive one dose of vaccine (0.5 mL) by intramuscular injection into the upper arm. The injection should be given into the opposite arm which blood was drawn. Subjects will be asked to stay at the site for 30 minutes after the vaccination to observe for immediate

reaction.

Subjects will be monitored for all adverse events during the first 4 weeks. Each subject will be instructed to record symptoms once each day (evening) on a diary card for 7 days. The following symptoms will be recorded: fever ($\geq 38^{o}$C), nasal congestion, cough, sore throat, muscle aches, headache, nausea, vomiting, malaise, eye redness, chest tightness, respiratory distress, and face edema. Moreover the local solicited adverse events, i.e., pain, swelling, redness, ecchymosis, and decrease limb mobility, at injection site will be recorded.

At 4 weeks after vaccination, subjects will be asked to return to the site for the second collection of blood samples. All adverse events during the first 4 weeks after the vaccination should be reported and recorded. The subject may return to the site according to her regular check-up schedule but the interval between the first and second blood sampling should be at least 3 weeks apart and no more than 5 weeks.

At the end of gestation period, blood samples from maternal subjects and from the cord blood will be collected, if applicable. Pregnancy outcome, including but not limited to the status of delivery, abortion and the date and/or the week of pregnancy of the event, will be recorded. The child information, including but not limited to the birth height and weight of the infant, sex of infant, Apgar score at 1 min and 5 min, and other information specified in the CRF, will be collected. All serious adverse events and adverse events of special interest will be followed until 8 weeks after the delivery.

5.2 Immunogenicity

Serum samples will be obtained prior to vaccination, and 4 weeks post-vaccination. Moreover, serum samples of maternal subject at the end of gestation period (delivery) and the cord blood sample will also be obtained, if applicable. Serum samples will be tested for anti-hemagglutinin (HA) antibodies by hemagglutination inhibition (HAI), and assays will be performed at Adimmune Corporation designated central laboratory. Subjects will be considered to be seronegative if subjects' serum HAI titer < 1:10. The seroconversion is defined as the post-vaccination serum at least 1:40 for whom had negative pre-vaccination or a four-fold or greater increase in HAI titers in subjects who had positive pre-vaccination serum.

All antigens used in this study will be formalin-inactivated whole viruses. The testing

antigens will be provided by the designated laboratory. The standard microtiter method will be used. Antibody titrations will be done in duplicate and the titer assigned to each sample will be the geometric mean of two independent determinations.

5.3 Safety

Safety data will consist of reactogenicity, serious and non-serious adverse events reported by the subject or observed by the investigator within 4 weeks after the vaccination, including 7 days after the dose of study vaccine. Vital signs will be performed at baseline and 4 weeks post-vaccination.

Reactogenicity events are pre-specified adverse events systematically recorded on diary cards (a grid of check boxes for each event and each day) during the immediate post-vaccination period by all participants. In general, reactogenicity events will be recorded for 7 days after vaccination. The selection of the events to be collected systematically is based on events expected to occur with wild-type influenza infection including fever (≥ 38°C), runny nose or nasal congestion, cough, sore throat, headache, muscle aches, vomiting, nausea and malaise. Furthermore, the local (injection site) reactions will also be evaluated that include soreness/pain, swelling, redness, ecchymosis and limitation of arm motion.

Adverse events of special interest, including influenza (with positive test for the presence of influenza), pneumonia (with positive X-ray or at least three of the six clinical criteria suggestive of pneumonia – reduced breathing frequency, dull percussion, local crepitation, bronchophony, temperature ≥ 38°C and thorax pain), heart failure (requires confirmation by cardiologist or at least three of five symptoms suggested of heart failure – edema, increased central venous pressure, pleural signs, enlarged heart, and dyspnea), stroke (diagnosed by a specialist), exacerbation of chronic pulmonary disease and other respiratory illness with fever, and all serious adverse events and adverse events of special interest on both maternal subjects and their infants will be monitored until 8 weeks after the devliery.

5.4 Schedule

The following table details the study schedule of procedures for study period:

	Visit 1			Visit 2	Visit 3	Follow-up
	Week 0	Week 1	Week 2~3	Week 4(1)	Delivery /Birth	8 Weeks after Delivery
Inform Consent	X					
Inclusion/Exclusion Criteria	X					
Vital Signs	X			X		
Weight and Height	X					
Medical History	X					
Pregnancy History/Outcome	X				X	
Serum Samples	X			X	X(2)	
Dosing	X					
Adverse Events	X---X				X(3)	X(3)
Reactogenicity events	X---------------X					
Diary Card	X---------------X					
Concomitant Medications	X---X					

(1): The subject will return to the site for blood sampling according to her regular check-up schedule. However, the interval between the first and second blood sampling should be at least 3 weeks apart and no more than 5 weeks.

(2): Including maternal serum (within 8 weeks after delivery) and cord serum

(3): Serious adverse events and adverse events of special interest should be collected

5.5 Serum Sample Randomization and Blinding

All serum samples sent to analysis laboratory will be under the blinded matter. On the serum samples used for antibody titrations there will be no information available regarding subject background data (e.g., age, pre- or post-serum). The serum will be re-labeled with serum number according to the randomization list for serum samples before being sent to the analysis laboratory. For each subject, there will be four randomization numbers allocated, one for the pre-vaccination serum sample (Visit 1), one for the post-vaccination serum sample (Visit 2, Week 4), one for the end of gestation period (Visit 3, delivery) serum sample from maternal subject (within 8 weeks after delivery) and one for the cord blood sample (Visit 3).

6. ADVERSE EVENTS

All reported adverse events within 4 weeks after the vaccination will be recorded as much as possible. Adverse event during the 30-minute observation period after the vaccination will be collected on site. Reactogenicity within the first 7 days after the vaccination will be recorded in the diary card. Specific adverse events of interest, i.e., Guillain-Barre syndrome, anaphylaxis, convulsions, encephalomyelitis, central nervous system demyelinating diseases, Bell’s palsy and vasculitis, will also be recorded in the CRF. Moreover, influenza, pneumonia, heart failure, stroke, exacerbation of chronic pulmonary disease and other respiratory illness with fever will also be recorded.

The term “adverse event” covers any sign, symptom, syndrome or illness that appears or worsens and is not of benefit to the subject during the period of observation in a clinical study. The term also covers laboratory findings (if applicable) or the results of other diagnostic procedures considered to be clinically relevant which:

- require additional diagnostic procedures not provided for in the study protocol
- require additional treatment measures not provided for in the protocol, or
- result in withdrawal of the subject from the study.

A study-emergent adverse event is defined as any event not present prior to signing informed consent or any event already present, which worsens in either intensity or frequency after signing informed consent. A treatment-emergent adverse event is defined as any event not present prior to exposure to study vaccine or any event already present, which worsens in either intensity or frequency following exposure to study vaccine.

The investigator is responsible for reporting all study-emergent serious adverse events and all treatment-emergent non-serious adverse events. If there is any doubt as to whether a clinical observation is a study or treatment-emergent adverse event, the event should be reported.

All adverse experiences must be rated on a 3-point scale of increasing severity, which defined as below:

Mild: Symptom barely noticeable to subject; does not influence performance or functioning. Prescription drug not ordinarily needed for relief of symptom but may be given because of personality of subject.

Moderate: Symptom of a sufficient severity to make subject uncomfortable; performance of daily activities influenced; subject is able to continue in study; treatment for symptom may be needed.

Severe: Symptom causes severe discomfort. May be of such severity that subject cannot continue the study. Severity may cause cessation of treatment with test drug; treatment for symptom may be given and/or subject hospitalized.

Baseline medical condition: It is not necessary to complete an adverse event form for adverse medical conditions present at baseline, which do not worsen in either intensity or frequency during the study. These adverse conditions should be adequately documented in the case report form and any other appropriate ancillary documents. Adverse medical conditions present at baseline, which become worse following exposure to study vaccine, should have an adverse event record completed.

Reactogenicity: events are pre-specified adverse events systematically recorded on diary cards (a grid of check boxes for each event and each day) during the immediate post-vaccination period by all participants. In general, reactogenicity events will be recorded for 7 days. The selection of the events to be collected systematically is based on events expected to occur with wild-type influenza infection. The events include fever (≥38℃), runny nose or nasal congestion, cough, sore throat, muscle aches, headache, vomiting, nausea and malaise. Furthermore, the local (injection site) reactions will also be evaluated that include soreness/pain, swelling, redness, ecchymosis and limitation of arm motion.

Serious adverse events: An adverse event which meets one or more of the following criteria is considered serious and must be reported in an expedited fashion:

- Results in death
- Is life-threatening
- Requires subject hospitalization or prolongation of existing hospitalization
- Results in persistent or significant disability/incapacity
- Is a congenital anomaly/birth defect
- Is medically important

Subjects experience the following events during the gestation period will also be reported as an serious adverse events: Guillain-Barre syndrome, anaphylaxis, convulsions, encephalomyelitis, central nervous system demyelinating diseases, Bell's palsy, vasculitis,

influenza, pneumonia, heart failure, stroke, exacerbation of chronic pulmonary disease and other respiratory illness with fever.

Deaths: In general, death should not be reported as an event. Death is viewed as an outcome of an event, rather than an event itself. In cases where the cause of death is unknown, “death” may be reported as an event on an initial serious adverse event form. However, every attempt should be made to submit a follow-up serious adverse event form identifying the probable cause of death when sufficient data are available.

Documentation process: All serious adverse events will be documented on a Serious Adverse Event forms, which will be completed by the study monitor with information provided by the investigator/study site. A copy of the Serious Adverse Event form will be filed with the subject’s case report form. All non-serious treatment-emergent adverse events will be reported in the case report form, which will be completed by the investigator/study site and are included in the subject’s case report form.

All serious adverse events, whether or not deemed vaccine-related or expected, during the study must be reported to designated representative immediately or within 24 hours (one working day) by telephone, telefax or electronic transmission.

A written report from the Investigator must be provided to the designed representative within 3 working days and is to include a full description of the event and sequel AE. This includes serious adverse events that occur anytime while enrolled in the trial. Written reports of all unexpected vaccine-related serious adverse events or authority-required serious adverse events must be submitted to the health authority and IRB in the hospital according to the regulated schedule of the health authority. Copies of each report will be kept in the investigator’s files, and adequate documentation notified of each serious adverse event.

7. STATISTICAL ANALYSIS

7.1 Sample Size Consideration

This is a descriptive study based on the use of 95% confidence intervals (CI), the sample size calculation is based on the expected precision of the results (extent of the 95% CI) and on the European recommendations for studies evaluating influenza vaccines to be used during each

influenza season (at least 50 subjects per group). Therefore, 60 maternal subjects are planned to be recruited.

For establishing the acceptability of the antibody response to H1N1, H3N2, and B for AdimFlu-S, the seroconversion rates for H1N1, H3N2, and B in the subjects receiving AdimFlu-S will be tested against a 40% lower bound for adult subjects.

According to the previous result, in the season of 2010-2011, the non-pregnant female (aged between 20 and 40) had the seroconversion rates were 84.6% against influenza A/H1N1 virus, 80.8% against influenza A/H3N2 virus and 38.5% against influenza B virus. Given 60 female subjects who receive AdimFlu-S will be evaluable for antibody titers, this study has at least 99% power to establish an acceptable seroconversion for A/H1N1 and A/H3N2, and 4.46% for B with 5% two-sided significance level.

7.2 Study Population

The immunogenicity analysis will be based on the intent-to-treat population, which is defined as all subjects who received a study vaccine and had evaluable serum sample after vaccination. All maternal subjects who received any amount of study vaccine will be included in the safety population.

7.3 Demographics

Descriptive statistics for demographic and subject characteristics prior to study vaccination including age, weight, height, gravidity and week of gestation will be reported.

7.4 Analysis of Immunogenicity

The immune response to the three vaccine viral strains is assessed by calculation of the geometric mean titers (GMT) of anti-HA antibodies, geometric means of post- to pre-vaccination antibody titer ratios (GMTR, with 95% confidence intervals), seroprotection rates (percentage of subjects with a titer >1:40 at 4 weeks after vaccination and at the end of gestation period), seroconversion rates. The seroconversion rate is defined as the percent of subjects with a pre-vaccination titer < 1:10 achieving a titer ≥ 1:40 or with pre-vaccination titer ≥ 1:10 having at least a four-fold rise at 4 weeks after vaccination and at the end of gestation period. As for the rate of seroprotection and seroconversion rate, the 95% exact (Clopper-Pearson) confidence interval will be provided as well.

European Agency for the Evaluation of Medicinal Products/Committee for Proprietary Medicinal Products (CPMP) immunogenicity criteria for licensed influenza vaccines specifies 70% seroprotection or 40% seroconversion, or a GMTR of 2.5 for individuals between 18 and 60 years of age.[12]

7.5 Analysis of Safety

The safety end points are the frequency, duration, and intensity of solicited adverse events during the 7 days after each vaccination and the incidence of serious adverse events during the study period.

The Medical Dictionary for Regulatory Activities (MedDRA) Terminology will be used to classify all adverse events with respect to System Organ Class (SOC) and preferred term. In the case of severity and relationship summaries, the most severe event and the most directly related event will be presented in summary tables in the case of multiple occurrences per subject.

8. ETHICAL AND LEGAL ASPECTS

8.1 Good Clinical Practice

The procedures set out in this study protocol, pertaining to the conduct, evaluation, and documentation of this study, are designed to ensure that the sponsor and investigator abide by good clinical practice (GCP). Compliance with these regulations also constitutes compliance with the ethical principles described in the current revision of the Declaration of Helsinki (Seoul, October 2008).

8.2 Delegation of Investigator Responsibilities

Responsibilities of the investigators conducting the trial in addition to those stated before are enumerated below:

- Obtain Institutional Review Board (IRB) approval to conduct the clinical trial.
- Provide sponsor with written documentation that the study protocol, any protocol amendments, and the informed consent form have received IRB approval.
- Provide sponsor with a list of IRB members, including their affiliations and

qualifications. As an alternative, a General Assurance number (as assigned by the Department of Health and Human Services) fulfills this requirement.

- Report to the IRB as required. The IRB must assume continued responsibility for the study and review the research on an annual basis.
- Maintain a file of all communications with the IRB on issues related to the clinical trial.
- Conduct the study according to the protocol, ICH - GCP guidelines and in accordance with the Declaration of Helsinki.

8.3 Subject Information and Informed Consent

Written informed consent must be obtained from all subjects prior to study participation. The informed consent form documents the information the investigator provides to the subject and the subject's agreement to participate. The investigator will fully explain the nature of the study, along with the aims, methods, anticipated benefits, potential hazards, and discomfort that participation might entail. The informed consent must be signed and dated by each subject or legal representative before entering the study and prior to the performance of any study specific procedures.

Investigational site must provide the sponsor (or designee) with a copy of the informed consent approved by that site's Institutional Review Board (IRB), or Ethics Committee (EC). The subject or the subject's legally authorized representative must sign the informed consent form. The original signed consent form will be retained in the subject's study records, and a copy will be provided to the subject. The sponsor will assure that each informed consent meets the basic elements of informed consent.

8.4 Confidentiality

Subject names will not be supplied to the sponsor. Only the subject number and subject initials will be recorded in the case report form, and if the subject name appears on any other document (e.g., laboratory report), it must be obliterated before a copy of the document is supplied to the sponsor. Study findings stored on a computer will be stored in accordance with local data protection laws. The subjects will be told that representatives of the sponsor or IRB may inspect their medical records to verify the information collected, and that all personal information made available for inspection will be handled in strictest confidence and in accordance with local data protection laws.

The investigator will maintain a personal subject identification list (subject numbers with the corresponding subject names) to enable records to be identified.

8.5 Protocol Amendments

The investigator is responsible for properly notifying the sponsor of protocol changes or revisions. The investigator without prior written authorization from the sponsor may make no changes to this protocol.

8.6 Approval of the Study Protocol and Amendments

Before the start of the study, the study protocol, informed consent document, and any other appropriate documents will be submitted to the independent ethics committee (IEC)/institutional review board (IRB) with a cover letter or a form listing the documents submitted, their dates of issue, and the site (or region or area of jurisdiction, as applicable) for which approval is sought. If applicable, the documents will also be submitted to the authorities, in accordance with local legal requirements.

Study medication can only be supplied to the investigator after documentation on all ethical and legal requirements for starting the study has been received by the sponsor. This documentation must also include a list of the members of the IEC/IRB and their occupation and qualifications. If the IEC/IRB will not disclose the names of the committee members, it should be asked to issue a statement confirming that the composition of the committee is in accordance with GCP. The IRB General Assurance Number may be accepted as a substitute for this list. Formal approval by the IEC/IRB should preferably mention the study title, study code, study site (or region or area of jurisdiction, as applicable), and any other documents reviewed. It must mention the date on which the decision was made and must be officially signed by a committee member.

Before the first subject is enrolled in the study, all ethical and legal requirements must be met.

The IEC/IRB and, if applicable, the authorities must be informed of all subsequent protocol amendments, in accordance with local legal requirements. Amendments must be evaluated to determine whether formal approval must be sought and whether the informed consent document should also be revised.

The investigator must keep a record of all communication with the IEC/IRB and, if applicable, between a coordinating investigator and the IEC/IRB. This also applies to any

communication between the investigator (or coordinating investigator, if applicable) and the authorities.

9. STUDY MONITORING AND AUDITING

Monitoring and auditing procedures developed by the sponsor or its designee will be followed, in order to comply with GCP guidelines. On-site checking of the case report forms for completeness and clarity, cross-checking with source documents, and clarification of administrative matters will be performed.

9.1 Study Monitoring

The sponsor designated representative will monitor this study in accordance with the Declaration of Helsinki (Seoul, October 2008). Responsibilities include study monitoring (adherence to protocol, review accuracy and completeness of records, source document checks); evaluation of study data (drug accountability, communication, and written records); monitoring of site facilities to ensure continued adequacy and AE monitoring. The designee will maintain regular contact with each investigational site, through telephone contact and on-site visits, to ensure that the investigational plan and DOH and other national and/or local regulations are followed. These contacts are also to ensure that timely and accurate data are submitted, that problems with inconsistent and incomplete data are addressed, and that the site facilities and management continue to be adequate for this study. Any questions regarding these matters should be addressed to the designee.

The designee will evaluate and summarize the results of each visit in written reports, identifying any repeated data problems with the investigator and specifying recommendations for resolution of noted deficiencies.

9.2 Source Documentation, Records Retention, and Availability

The investigator must maintain detailed, primary records (i.e., source documents) of the data for each subject screened and treated to study treatment. Examples of source documents may include, but are not limited to: signed informed consent forms, office or clinic records, hospital records, progress notes, diagnostic test results, telephone contact reports, laboratory reports, consultant’s opinions/findings, referral letters, study drug inventory records.

The investigator must make study data accessible to the monitor, sponsors, or other authorized representatives of the sponsors and regulatory agency inspectors. A file for each subject must be maintained that includes the signed informed consent form and copies of all source documentation related to that subject. The investigator must ensure the availability of source documents from which the information on the CRF is derived.

9.3 Source Data Verification and On-Site Audits

Regulatory authorities, the IEC/IRB, and/or the sponsor’s clinical quality assurance group may request access to all source documents, case report forms, and other study documentation for on-site audit or inspection. Direct access to these documents must be guaranteed by the investigator, who must provide support at all times for these activities.

10. REFERENCE

1. Neuzil, K.M., Reed, G.W., Mitchel, E.F., Simonsen, L., Griffin , M.R. 1998. "Impact of influenza on acute cardiopulmonary hospitalizations in pregnant women." *American Journal of Epidemiology*, 148:1094-102.

2. Hartert, T., Neuzil, K., Shintani, A., Mitchel, E., Snowden, M., Wood, L., Dittus, R., Griffin , M. 2003. "Maternal morbidity and perinatal outcomes among pregnant women with respiratory hospitalizations during influenza season." *American Journal of Obstetric Gynecology*, 189:1705-12.

3. Centers for Disease Control and Prevention. "Prevention and control of influenza with vaccines: recommendations of the Advisory Committee on Immunization Practices (ACIP), 2010." *MMWR 2010*; 59 (No. RR-8): 1-63.

4. ACOG Committee on Obstetric Practice. 2004. Influenza vaccination and treatment during pregnancy. ACOG Committee Opinion No. 305, November 2004. *Obstetric Gynecology*, 104 (5 Pt 1):1125-6.

5. Black, S., Shinefield, H., France, E., Fireman, B., Platt, S., Shay, D. 2004. Vaccine Safety Datalink Workgroup. "Effectiveness of influenza vaccine during pregnancy in preventing hospitalizations and outpatient visits for respiratory illness in pregnant women and their infants." *American Journal of Perinatology*, 21:333-9.

6. Zaman K, Roy E, Arifeen S, Rahman M, Raqib R, Wilson E, et al. "Effectiveness of maternal influenza immunization in mothers and infants." *N Engl J Med*. [10.1056/NEJMoa0708630]

7. Heinonen, O.P., Shapiro, S., Monson, R.R., Hartz , S.C. , Rosenberg , L., Slone, D. 1973. "Immunization during pregnancy against poliomyelitis and influenza in relation to childhood malignancy." *International Journal of Epidemiology*, 2:229-35.

8. Deinard, A.S., Ogburn, P. 1981. A/NJ/8/76 influenza vaccination program: effects on maternal health and pregnancy outcome. *American Journal of Obstetric Gynecology*, 140:240-5.

9. Munoz, F., Greisinger, A., Wehmanen, O., Mouzoon, M., Hoyle, J., Smith, F., Glezen, W. 2005. Safety of influenza vaccination during pregnancy. *American Journal of Obstetric*

Gynecology, 192:1098-106.

10. Campbell DS, Rumley MH. Cost-effectiveness of the influenza vaccine in a healthy, working-age population. *J.Occup Environ Med*. 1997; 39:408-414.

11. Doherty PC, Turner SJ, Webby RG, Thomas PG. Influenza and the challenge for immunology. *Nat Immunol.* 2006; 7(5):449-455.

12. EMEA. Harmonization of requirements for influenza vaccines. (CPMP/BWP/214/96) 1997.

13. World Health Organization. Recommended viruses for influenza vaccines for use in the 2010-2011 northern hemisphere influenza season. February 2010. http://www.who.int/csr/disease/influenza/201002_Recommendation.pdf
