# Supplementary material for: The Safety and Immunogenicity of Trivalent Inactivated Influenza Vaccination: A Study of Maternal-Cord Blood Pairs in Taiwan
Source: PLoS One. 2013 Jun 6;8(6):e62983. doi: 10.1371/journal.pone.0062983 (PMC3675132; doi:10.1371/journal.pone.0062983)
Supplement: Text S1 — The approval of the study by the Research Ethics Committee A of the National Taiwan University Hospital. (PDF) [file pone.0062983.s003.pdf]

檔　號：
發文方式：郵寄
保存年限：

國立臺灣大學醫學院附設醫院　函

地址：10002台北市中山南路7號
承辦人：林景雅
電話：02-2312-3456分機63594
傳真：02-2395-1950
電子信箱：ntuhrec@ntuh.gov.tw

11051
臺北市信義區信義路4段415號3樓之1

受文者：晉加股份有限公司

發文日期：中華民國100年11月3日
發文字號：校附醫倫字第1003704093號
速別：普通件
密等及解密條件或保密期限：普通
附件：如文

主旨：有關本院婦產部李建南醫師所主持之「三價非活性流感疫苗(安定伏)於孕婦之免疫抗原性及安全性研究」（案件編號：201109006MA；計畫書編號：FLU11T12P）藥品已有衛生署許可證臨床試驗計畫案，通過本院A研究倫理委員會第21次會議審查，符合研究倫理規範，惟應依說明段辦理。隨函檢附「臨床試驗許可書」乙份，請　查照。

說明：

一、依據本院100年10月21日A研究倫理委員會第21次會議決議辦理，請自行上網查詢會議紀錄：（網址：http://www.ntuh.gov.tw/RECO）。

二、本臨床試驗核准之有效期限為1年，計畫主持人應於到期前的1個月提出持續審查申請表，本案需經持續審查，方可繼續執行。

三、本臨床試驗計畫若需變更、暫停執行、中途終止或結束時，主持人應向本會提出審查申請。

四、敬請於收到本許可公文後，備妥相關文件俾便進行後續之臨床試驗合約書簽署作業，詳細內容敬請參照研究倫理委員會網頁(有贊助者之臨床試驗計畫試驗經費帳戶之設立及作業流程)說明：http://bme.mc.ntu.edu.tw/clinical_trial/

CTC/CTCSA.htm。

五、計畫主持人及研究團隊應遵循之相關研究倫理規範，請參閱研究倫理委員會網頁http://www.ntuh.gov.tw/RECO，並遵照執行;臨床試驗執行期間，請確實依據「藥品優良臨床試驗準則」之相關規定辦理；為符合「藥品優良臨床試驗準則」結案查核作業，請計畫主持醫師保存所有文件備查。

六、隨函另檢附SAE通報須知、臨床研究重要訊息通知單，請依計畫需要辦理相關事宜。

正本：國光生物科技股份有限公司、晉加股份有限公司、本院婦產部李建南醫師
副本：本院臨床試驗用藥管理中心、研究倫理委員會(皆含許可書附件)

院長 陳明豐

國立台灣大學醫學院附設醫院A研究倫理委員會

Research Ethics Committee A
National Taiwan University Hospital
7, Chung-Shan South Road, Taipei, Taiwan 100,R.O.C
Phone: 2312-3456 Fax: 23951950

臨床試驗許可書

許可日期：2011 年 11 月 02 日

倫委會案號：201109006MA

計畫名稱：三價非活性流感疫苗(安定伏)於孕婦之免疫抗原性及安全性研究。

部門/計畫主持人：婦產部　李建南醫師

計畫文件版本日期：【*計畫書：Version 1.0/2011-08-08、同意書：Version 1.2/ 31 Oct 2011、個案報告表：Version 1.0/ 2011-08-08、中文摘要：Version 1.1/ 2011-09-26、受試者招募文宣：Version 1.2/ 31 Oct 2011、受試者日誌：Version 1.0/2011-08-08*】

上述計畫業經 2011 年 10 月 21 日本院 A 研究倫理委員會第 21 次會議審查同意，符合研究倫理規範。本委員會的運作符合優良臨床試驗準則及政府相關法律規章。

本臨床試驗許可書之有效期限為 1 年(自 2011 年 11 月 2 日起至 2012 年 11 月 1 日止)。

計畫主持人須依國內相關法令規定通報嚴重不良反應事件，並應於到期前的 1 個月內提出持續審查申請表，本案需經持續審查，方可繼續執行。

主任委員

何弘能

Clinical Trial Approval
National Taiwan University Hospital

Date of approval: Nov 02, 2011

NTUH-REC No.：201109006MA

Title of protocol：The safety and immune response to influenza vaccination in pregnant women.

Department/ Principle Investigator：Department of Obstetrics & Gynecology / Dr. Chien-Nan Lee

Version date of documents:【***Protocol: Version 1.0/2011-08-08; ICF: Version 1.2/ 31 Oct 2011; CRF: Version 1.0/ 2011-08-08; Chinese synopsis: Version: Version 1.1/ 2011-09-26; Patient Recruitment Poster: Version 1.2/ 31 Oct 2011; Diary Card: Version 1.0/2011-08-08***】

The protocol has been approved by the 21th meeting of Research Ethics Committee A of the National Taiwan University Hospital on Oct 21, 2011. The committee is organized under, and operates in accordance with, the Good Clinical Practice guidelines and governmental laws and regulations.

The duration of this approval is one year (from Nov 2, 2011 to Nov 1, 2012). The investigator is required to report Serious Adverse Events in accordance with the governmental laws and regulations, and apply for a continuing review within one month prior to the approval expiration date.

Hong-Nerng Ho

Hong-Nerng Ho, M.D.
Chairman
Research Ethics Committee A

2011~2012 季節性流感疫苗臨床試驗 開始了

"安定伏"季節性流感疫苗
預防流感人體臨床試驗

李建南
2011.10.31

本季節性流感疫苗係依今年(2011~2012)世界衛生組織建議更新之病毒株組成 (A/California/7/2009 (H1N1)， A/Perth/16/2009 (H3N2) 及 B/Brisbane/60/2008)

本研究之目的為評估懷孕婦女接種季節性流感疫苗(安定伏)後其免疫性及安全性。

流感疫苗為不活性的疫苗，因此，您應不會因接種疫苗而感染。

若您是<u>**年滿18歲之懷孕婦女**</u>，並沒有對疫苗過敏及對雞蛋或雞蛋蛋白質過敏之病史。且在過去六個月內，未接種過其它流感疫苗並也沒有感冒發燒的現象。

若您參與本研究，您將接受一劑的國光"安定伏"季節性流感疫苗接種。並於加入試驗後，須配合醫師指示回診，進行相關檢驗及追蹤。試驗執行期間為加入試驗至您生產後8週。

國立臺灣大學醫學院附設醫院研究倫理委員會

凡有意願參與此一臨床試驗者，
請向 台大醫院 婦產科聯絡。(試驗主持人:李建南醫師)

■聯絡方式：自即日起 分機:70919

◆ 研究護士 待聘中 聯絡電話：

試驗機構地址：台北市中正區中山南路8號 (台大醫院兒童醫院9樓 母胎兒中心)

台大醫院

Version 1.2 / 31 Oct 2011
